# The locus for an inherited cataract in sheep maps to ovine chromosome 6

**Published:** 2012-05-31

**Authors:** Gareth R.S. Wilson, James D. Morton, David N. Palmer, John C. McEwan, Karl Gately, Rayna M. Anderson, Ken G. Dodds

**Affiliations:** 1 Faculty of Agriculture and Life Sciences, Lincoln University, Lincoln, New Zealand; 2 Invermay Agricultural Centre, AgResearch, Mosgiel, New Zealand

## Abstract

**Purpose:**

Cataracts are an important cause of blindness in humans but there are few large animal models available. One of these animal models is Ovine Heritable Cataract, a bilateral cortical cataract which develops after birth. This cataract has been used as a model for human cataracts in drug trials, but the gene responsible for the cataract trait is unknown. A genetic test for cataract would improve the efficiency of the model by predicting which animals would develop cataracts. Identifying the genetic basis of the cataract would indicate its relevance to human cataract.

**Methods:**

A genome scan was performed on 20 sheep chromosomes, representing 86% of the genome, to determine the position of the cataract locus. Additional microsatellite markers were tested on chromosome 6 using a larger pedigree. Fine mapping was performed using a breakpoint panel of 36 animals and novel microsatellite markers taken from the bovine genome assembly. All exons of the candidate gene nudix (nucleoside diphosphate linked moiety X)-type motif 9 (*NUDT9*) were sequenced in normal and affected sheep.

**Results:**

Significant linkage was found between cataract status and markers on chromosome 6. Linkage analysis on the larger pedigree showed the most likely position of the cataract locus was between 112.3 and 132.9 cM from the centromere. During fine mapping, *NUDT9* was considered as a positional candidate for the cataract gene because it was located within the linked interval and is expressed in the lens. The gene was ruled out as the cataract gene after extensive genotype analysis, but a single nucleotide polymorphism (SNP) inside it provided a useful restriction fragment length polymorphism (RFLP) marker for further fine mapping. Twelve new markers were found and used to map the cataract locus to between 131.1 and 131.8 cM from the centromere.

**Conclusions:**

A region of ovine chromosome 6 strongly linked to cataract has been identified, and a genetic test for cataract based on a SNP within this region has been developed. The best candidate gene within this region is AF4/FMR2 family, member 1 (*AFF1*), the mouse equivalent of which is associated with an inherited cataract.

## Introduction

Opacities in the lens are referred to as cataracts [1] and are one of the most common causes of blindness in humans. Cataract formation usually involves the aggregation of lens proteins or the disruption of lens fiber structure. This can be caused by specific insults such as UV light or by genetic defects, but most human cataracts occur after the age of 40 via mechanisms which are still poorly understood [2]. Several animal models have been used to study cataract formation mechanisms. These are mostly rodents with hereditary cataracts, however rodents have small lenses that are hard to dissect and need to be pooled for analysis [3]. One example of an inherited cataract in a large animal that may be a more convenient experimental model is Ovine Heritable Cataract (OHC).

OHC was first reported in a flock of New Zealand Romney sheep [4]. Most lenses develop cortical opacities 1–2 months after birth, with the entire lens becoming opaque by 10 months [5]. The condition was found to be inherited in a dominant manner, and easily controlled by culling, so therefore not an important genetic defect from an economic perspective. However, fewer affected offspring are produced from affected sheep than would be expected from simple dominant inheritance.

OHC is convenient as a model for human cataracts because sheep lenses are twice as large as human lenses and are relatively easy to dissect and manipulate. OHC sheep have been used in trials of cataract prevention drugs [5,6]. One difficulty with OHC as an experimental model is that the genetic basis of the condition is unknown. Affected animals can only be identified by observing the development of the cataract, and homozygotes can only be identified by the proportion of affected offspring.

Identifying the OHC gene would enable genetic testing to determine which lambs would develop cataracts and make drug trials more efficient. It would also indicate the mechanism of cataractogenesis in OHC and its relevance to human cataracts. In this study, a genome scan was performed on a pedigree containing sheep affected by OHC and related normal sheep. The results of the genome scan were used with an annotated bovine genome assembly to indicate candidate genes for OHC. One candidate was nudix (nucleoside diphosphate linked moiety X)-type motif 9 (*NUDT9*), which codes for an ADP-ribose phosphorylase [7] known to be expressed in the lens [8]. This could cause cataract through ADP-ribose accumulation leading to either glycoxidation [9] or interference with calcium homeostasis [10]. AF4/FMR2 family, member 1 (*AFF1*) was also within the linked region and a mutation in its homolog in mice leads to a dominant bilateral cataract that develops after birth [11].

## Methods

### Animal Ethics

All animal procedures in this study were authorised by the Lincoln University Animal Ethics Committee, Lincoln, New Zealand, in compliance with the New Zealand Animal Welfare Act, 1999.

### Pedigree material

Affected Romney rams were outcrossed with normal Coopworth ewes and the affected offspring were outcrossed to normal unrelated sheep and also crossed with each other.

A veterinary ophthalmologist identified animals with cataracts by examining them with a slit lamp. The lenses were scored on a scale from 0 (no cataract) to 7 (mature cataract with lens resorbing) as described earlier [6]. All lambs had their lenses examined at least twice and up to six times. The inheritance of the cataract trait was studied by recording the proportion of affected offspring from matings between affected rams and normal Coopworth ewes, and also between pairs of affected animals. Litter sizes and rates of death before eye examination were studied to determine whether a lethal homozygote effect existed.

An affected ram (38/03) and 41 of his offspring with normal ewes were selected for sampling of DNA for genotyping and locus localization. Twenty of the offspring were affected by cataracts and 21 were unaffected. DNA was extracted from venopuncture blood samples of 5–8 ml by the guanidine isothiocyanate method [12].

After genotyping (see below), linkage analysis was performed on this set of samples. Additional animals were then tested using the same procedure for markers on the chromosome showing significant linkage. These animals included 3 more affected rams and 229 of their offspring with normal ewes, and 6 affected ewes and 9 of their offspring with the 4 affected rams. A breakpoint panel consisting of 36 animals that had recombined in the region linked to OHC was assembled from these samples, using the results of marker genotyping and linkage analysis. Before linkage analysis of the breakpoint panel, 10 affected ancestors of the animals in the panel were also genotyped, using preserved DNA samples. The total number of animals genotyped was 296.

### Marker genotyping

The samples from the first ram and his 41 offspring were genotyped with a set of 149 microsatellite markers over 20 chromosomes. Primers and PCR conditions for these markers are available at the Australian Sheep Gene Mapping Web Site. The first 10 chromosomes were selected because their homologs in humans or mice contained loci associated with dominant cataract. The next 10 were the largest chromosomes remaining. As a strong linkage was found on one of these, the remaining six chromosomes (20, 21, 22, 23, 24, and 25) were not analyzed. The average separation of the markers in the initial analysis was 20 cM. On the larger pedigree (descended from the four rams), a further 4 markers on chromosome 6 were genotyped, as well as the original 7. The 36 animals in the breakpoint panel and their 10 ancestors were tested for 12 additional markers on chromosome 6 (Table 1). Eleven were microsatellites discovered from the bovine genome assembly and one was a single nucleotide polymorphism (SNP; c.989G>A) in the coding sequence of *NUDT9*, a gene in the linked region.

**Table 1:** Fine mapping markers.

**Marker**	**Forward Primer**	**Reverse Primer**	**Tm (°C)**
BMS1509192	GAAGCAACCTAAGTGTCCATCA	ATCCATGTTTTTGCAAGTGG	TD
BMS1509996	TGTGAAGAGGAAAAGGCATCA	CTCCTCACGGGCAAGTAAGT	TD
BMS1510292	CGCAGCGAGGAAAATGTTTA	GGTCAGAATGACCTGGTCTCA	TD
BMS1512327	TTTGCCCAAGAGATGGATTG	GCCTGGAAGACTACGGTCTG	TD
BMS1512786	GATGGGAGAGCTGGAGACTG	CCTGGACTCTGCTCATCAAA	TD
BMS1512958	TGTATTTTGGGGGATGGAAA	ACATGCCTTGAGGCCAAA	TD
BMS1513060	GGAGAAAATTGAGGCAGCTT	CTGCAGCTCTGCAAAACCTA	TD
BMS1513944	GCACTGCGCTATTGGTGTTA	TTGTGGGGACACAGTTCAAA	TD
T3S9	TTTCCAGGCACTTTGTTTCC	AGGGCTTATAGGCGTGATGA	64.3
T4S11	CACTTCTGTGCTGCTGTGGT	TCAATCATCCTCCAAACACAA	61.2
T6S8	TGTGAAAGATGTCACAAATCACTG	GGAAGATTCCCTGGAGAAGG	57
c.989G>A	CCTTGCCTTAGAAGCTGGAG	GCAGCCAGTCTCAGAGTCCT	59

Primers and annealing temperatures for novel OAR chromosome 6 markers are shown. TD indicates a touchdown PCR, described in the marker genotyping section of the Methods.

Eight of the microsatellite markers were amplified using a touchdown PCR cycle. Each PCR reaction mixture consisted of a 1:10 dilution of venopuncture DNA extract (at least 0.6 μg per mL), 200 μM dNTP mix (Life Technologies, Carlsbad, CA), 1 μM of each primer, 5 mM MgCl_2_, and 0.5 units per μl of Platinum *Taq* DNA polymerase (Life Technologies). The final volume of each mixture was 10 μl. The PCR cycle involved an initial denaturation, 94 °C for 5 min, followed by 5 cycles of 94 °C for 30s, 60 °C for 30 s, and 72 °C for 40 s. The 5 cycles were repeated with annealing temperatures of 58 °C, 56 °C, 54 °C (twice), and 50 °C, for a total of 30 cycles. There was a final elongation step, 72 °C for 45 min.

The PCR cycle for T3S9 had annealing temperatures of 61.2 °C for 7 cycles, followed by 64.3 °C for 23 cycles, with a final elongation step of 7 min. The concentration of MgCl_2_ was 1 mM. All other PCR conditions were as described above. For c.989G>A, T4S11, and T6S8, the PCR conditions were the same as for T3S9, except that the annealing temperatures (Table 1) were the same over all 30 cycles and the final elongation step was 10 min.

PCR products were visualized after electrophoresis on a 2% agarose gel containing ethidium bromide, 90V 1 h, to confirm amplification and the approximate size of the products.

For genotyping, the PCR conditions were modified to produce fluorescently labeled products as described [13], and the products were visualized with an ABI3730 DNA Analyzer (Life Technologies, Carlsbad, CA), using capillary electrophoresis with fluorescence detection. Allele sizes were determined using GeneScan™ –500 LIZ^®^ Size Standard and GENEMAPPER Version 3.7 software (Life Technologies) was used with the output of the DNA analyzer to assign genotypes.

For c.989G>A, the majority of animals were genotyped by restriction fragment length polymorphism (RFLP) analysis using the restriction enzyme HpyCh4IV (New England Biolabs, Ipswich, MA), with the remainder genotyped by sequencing of a PCR product surrounding c.989G>A. Restriction digests were performed on 5 μl of each PCR product. Each digest mixture contained 2.5 μl of 10× buffer, 0.25 μl (2.5U) of HpyCh4IV, and 17.25 μl of dH_2_O to bring the total volume to 25 μl. The mixtures were incubated, 37 °C 1 h, and visualized after electrophoresis on a 2.5% agarose gel containing ethidium bromide, 90 V for 30 min.

### Linkage analysis

The CRIMAP software package [14] was used to perform linkage analysis on the marker genotypes. The first set of genotypes was treated as a single family with one sire, 26 dams, and 41 offspring. All dams were entered as homozygous normal for the OHC locus and unknown for all the markers. All affected animals were entered as heterozygous and all unaffected animals as homozygous normal for the OHC locus. Since OHC shows incomplete penetrance (see below), some of the unaffected animals with affected parents will have the affected allele of the OHC locus. However, since cataract is rare in sheep, unrelated normal ewes from the general Coopworth population are very unlikely to have the affected allele. Some affected animals with two affected parents may be homozygotes for the affected allele, but there is no evidence of this at present.

The larger set of animals was treated as a single family with 4 related rams, 6 affected ewes, 136 unrelated normal ewes and 279 offspring. Affected ancestors of these sheep were added to the family and scored as unknown for all markers if no DNA was available from them. All affected animals were again entered as heterozygous for the OHC locus, including affected animals with two affected parents. One-third of the latter would be expected to be homozygous assuming dominant inheritance, but since none have been identified as homozygotes they must all be entered as heterozygotes. When more markers were tested on the breakpoint panel, these genotypes were added to the input file and animals not in the breakpoint panel were scored as unknown for these markers. No extra information on mode of inheritance, penetrance, or allelic frequencies was included in the pedigree files.

Two-point logarithm of odds (LOD) scores between the OHC locus and each marker were generated by the CRIMAP software, along with the most likely interval between the markers to contain the OHC locus for each set of genotypes. A LOD score of 3 was considered to be significant. Multipoint analysis was performed using MultExclude, a SAS program developed by one of the authors (K.G.D.).

The bovine homolog of the linked region was examined using the bovine genome assembly Btau4.0 to find candidate genes for cataract. This was the most reliable bovine genome assembly at the time the investigation was undertaken. It is annotated with known bovine genes and homologs of human genes identified by comparison with the human genome assembly. The sheep genome assembly available at the time, Oar1.0, was not sufficiently reliable for a comprehensive search for candidate genes. However, Oar1.0, Btau4.0, and the latest human genome assembly hg19 were used to compare the order of genes in the linked region across species.

### NUDT9

*NUDT9* was identified as a candidate gene for OHC because it was located within the region given by the early linkage analysis and known to be expressed in the lens [8]. To determine if a mutation in *NUDT9* was associated with OHC, the coding sequence of *NUDT9* was sequenced in 6 animals, 3 affected and 3 normal. Total cDNA was prepared from RNA extracted from venopuncture blood, using Superscript III reverse transcriptase (Life Technologies) and a poly-T primer. The cDNA was used as the template for PCRs with several pairs of primers (Table 2) designed to amplify the entire *NUDT9* coding sequence. The first forward primer was located before the start codon and the last reverse primer was located after the stop codon, so a small non-coding sequence was amplified at each end. The primers inside the coding sequence were located in positions that generated overlaps between each adjacent pair of sequences.

**Table 2:** NUDT9 sequencing primers.

Primer	Sequence	Tm (°C)	Product size (bp)
NUDT9F	TCCTGGGGAAGACTTTAGCC	60.6	996
NUDT9R	CATCTCGTTCTCTGCCACA	60.0	
StartF	GCGGCTCAAGCTAAGAGCTA	60.0	283
StartR	TGCGCTGAACTTTTGAACCT	60.9	
GapF	TTTCTGTCTTGGCTGGACCT	59.8	378
GapR	TCTTTTCAGTGTGGCGCTAA	59.6	
EndF	ATGCTGGAAAGGTGAAATGG	59.9	168
EndR	GGCTTTTGGCTTACATGGAG	59.7	
NUDT9BLDF	GCTCTTCAGCCAGGAACATC	60.0	277
NUDT9BLDR	GCAGCCAGTCTCAGAGTCCT	59.7	
NUDT9DNA2F	CCTTGCCTTAGAAGCTGGAG	59.2	151
NUDT9DNA2R	GCAGCCAGTCTCAGAGTCCT	59.7	

Sequences, melting temperatures, and predicted product sizes from bovine sequence are shown. NUDT9BLDR and NUDT9DNA2R are identical, but were generated from separate runs of the primer design software.

The PCR products were purified by electrophoresis on a 2% agarose gel and extracted with an Axyprep DNA gel extraction kit (Axygen Biosciences, Union City, CA). Purified PCR products were sequenced by the Genome Service (Massey University, Palmerston North, New Zealand), using a BigDye^TM^ Terminator Version 3.1 Ready Reaction Cycle Sequencing Kit (Life Technologies) and an ABI3730 capillary instrument.

The sequences of all six animals were analyzed to find polymorphisms that correlated with cataract status, allowing for incomplete penetrance. When these were identified, primers were designed to amplify these polymorphisms from both cDNA and genomic DNA.

Additional animals were genotyped for the correlated polymorphisms using these primers and the same sequencing procedure.

The restriction enzyme HpyCh4IV has the recognition site ACGT, with the cleavage site between A and C. The c.989G>A SNP is the third base (G) of an HpyCh4IV recognition site inside the *NUDT9* coding sequence. Therefore the enzyme cleaves the sequence surrounding the polymorphism only when it is in the G form, which is associated with normal animals. HpyCh4IV was used to generate c.989G>A genotypes using RFLP analysis, as described above.

The total number of animals genotyped for this polymorphism was 238. The cataract statuses of the animals were compared with the genotypes to determine if the polymorphism was a candidate for the cataract mutation. As described above, this polymorphism was also included as a marker in the linkage analysis.

To test c.989G>A as a genetic marker for cataract, 81 additional lambs were genotyped using the RFLP test and examined to determine their cataract status. The c.989G>A genotypes were used to predict the cataract status of the lambs, and the predictions checked against the actual phenotypes. The genotypes were not available to the veterinarian who scored the cataract statuses of the lambs. All lambs with the GG genotype were predicted to be normal, and all lambs with the AG or AA genotypes were predicted to be affected.

## Results

### Progression of cataract

The lambs were first examined for cataracts after weaning, typically at two months of age.

**Figure 1:**
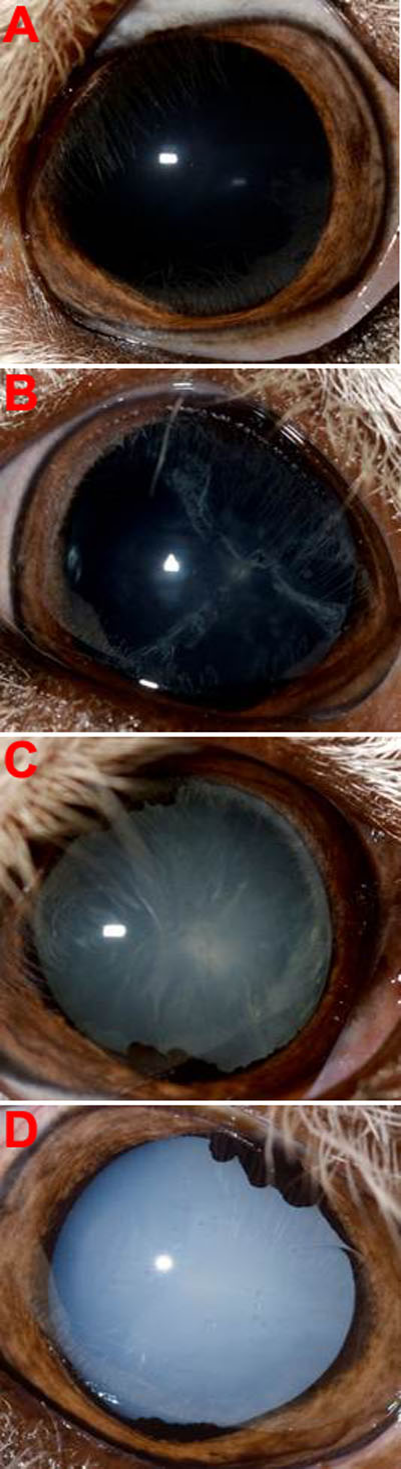
Examples of cataract progression. **A**: A normal lamb eye without cataract. **B**: A stage 2 cataract with opacities at the suture lines. **C**: A stage 4 cataract with more than a third of the lens surface opaque. **D**: A stage 6 or mature cataract.

Most of those with cataracts had scores of 1 or 2 (Figure 1B) at this age. These lenses show opacities that are often associated with the suture lines. Over the next 4 to 6 months the opacity spreads to cover the entire lens as mature cataracts (Figure 1D) and eventually some would show signs of resorbing the lens [6].

### Inheritance

Investigation of the inheritance of the cataract trait showed that it could be inherited from either parent by both male and female animals [15]. In both animals with one affected parent and with two affected parents, there was no significant difference in cataract rates between males and females.

In animals with one affected and one unaffected parent, the proportion of cataracts was 0.40±0.02, compared to the expected 0.5 for autosomal dominant inheritance (Table 3). With two affected parents the proportion was 0.62±0.03, compared to the expected 0.75 (Table 3). A χ^2^ goodness of fit test shows that the difference between expected and actual results is significant in both cases (p<0.0001). A weighted average of the two sets of animals gives a penetrance for the cataract trait of 0.81±0.04.

**Table 3:** Lamb cataract statuses.

**Lamb Dams**	**Normal**	**Affected**	**Total**	**Proportion affected (expected)**
**Normal Dam**				
Female	244	144	388	0.37 (0.5)
Male	220	166	386	0.43 (0.5)
Total	464	310	774	0.40 (0.5)
**Affected Dam**				
Female	52	81	133	0.61 (0.75)
Male	48	79	127	0.62 (0.75)
Total	100	160	260	0.62 (0.75)

Numbers of offspring with an affected sire and both normal and affected dams are shown, with proportions affected.

Litter sizes are not significantly different between offspring with two affected parents and with only one. However, a χ^2^ independence test shows that death rates are significantly higher in offspring of two affected parents (0.10±0.02, 305 animals) versus one affected and one normal parent (0.052±0.008, 714 animals).

### Linkage analysis

**Figure 2:**
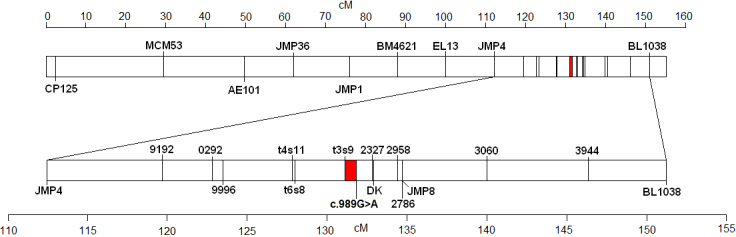
Schematic diagram of sheep chromosome 6. The rectangle at the top shows the entire chromosome, with the centromere on the left and the telomere on the right. Vertical lines show the positions of markers used in linkage analysis. The linked region from the latest CRIMAP analysis is shown in red. The upper scale shows distance along the chromosome in centiMorgans. The lower rectangle shows an expanded view of the region between markers JMP4 and BL1038. The linked region is also shown in red. The lower scale shows distance along the expanded region of the chromosome in centiMorgans. BMS150 and BMS151 markers are indicated by the last four digits of the marker name. Marker DK1183A is represented by DK.

The 20 chromosomes on which markers were genotyped were OAR1–19 inclusive, and 26. The highest LOD score between any marker and the OHC locus was 4.24, from marker JMP8 on OAR6. Two other markers on chromosome 6 also had significant LOD scores. These were JMP12 with a LOD score of 3.77, and BL1038 with a LOD score of 3.05. The highest LOD score on the other chromosomes was 1.12 from marker LS55 on OAR7. The ALL option of CRIMAP determined the most likely position for the OHC locus on chromosome 6 to be between JMP4 and JMP8, equivalent to between 112.3 and 135.5 cM from the centromere (Figure 2).

Fine mapping was performed using the larger pedigree of 296 animals. The highest LOD score was 38.71 from marker DK1183A. Five other microsatellite markers (BM4621, EL13, JMP4, JMP8, and BL1038) also had highly significant linkage to OHC, with LOD scores greater than 7. CRIMAP calculated the most likely position for the cataract locus to be between markers JMP4 and DK1183A, equivalent to between 112.3 and 132.9 cM from the centromere.

The breakpoint panel was genotyped for 12 new markers, reducing the most likely region for the cataract locus to between markers t3s9 and c.989G>A. This is equivalent to between 131.1 and 131.8 cM from the centromere (Figure 2).

**Figure 3:**
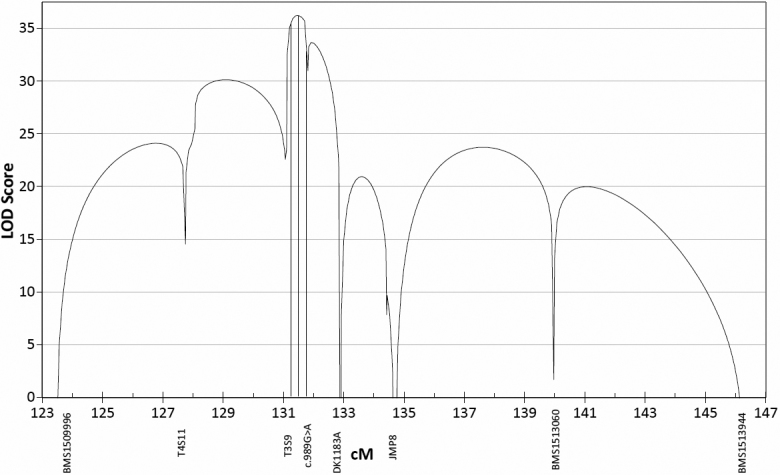
Multipoint linkage analysis of the candidate OHC region. This analysis is based on the genotypes of 11 markers in 296 animals, plus the genotypes of 12 more markers in 48 of the 296 animals. The three vertical lines indicate the 1-LOD support interval and the highest LOD score within it. Markers are indicated by vertical text along the bottom, and some have been omitted.

Multipoint analysis gave a 1-LOD support interval of 131.3 to 131.7 cM (Figure 3).

CRIMAP was also used to calculate two-point LOD scores representing the genetic linkage between markers on chromosome 6 and the cataract locus (Table 4). The markers genotyped only on the breakpoint panel showed smaller LOD scores than the other markers, because fewer genotypes were available and therefore fewer informative meioses.

**Table 4:** Marker two-point LOD scores.

**Marker**	**centiMorgans**	**LOD**
CP125	2.60	0.00
MCM53	29.70	0.50
AE101	49.90	−0.00
JMP36	62.20	0.28
JMP1	76.10	1.29
BM4621	88.10	10.91
EL13	100.10	12.10
JMP4	112.40	15.14
BMS1509192	119.69	0.00
BMS1510292	122.76	−0.00
BMS1509996	123.46	−0.00
t4s11	127.75	0.08
t6s8	128.06	−0.00
t3s9	131.11	3.94
c.989G>A	131.81	5.55
BMS1512327	132.82	0.30
DK1183A	132.90	41.82
BMS1512958	134.42	26.91
BMS1512786	134.68	3.30
JMP8	134.70	2.82
BMS1513060	139.97	3.17
BMS1513944	146.28	0.35
BL1038	151.20	7.80

Positions are given in centiMorgans from the centromere of sheep chromosome 6. LOD scores are calculated by two-point analysis between each marker and the OHC locus, using CRIMAP and the latest genotypes and cataract statuses of the sheep flock.

A comparison of gene positions using sheep, bovine and human genome assemblies showed no evidence of any rearrangement in the linked region between sheep and cattle. The homologous region in humans was inverted, but the relative order of the genes was identical.

### NUDT9

Five polymorphisms were identified in the *NUDT9* coding sequence, c.153G>A, c.855G>T, c.946G>A, c.989G>A, and c.992T>G. Polymorphisms c.855G>T and c.989G>A were correlated with cataract status, allowing for incomplete penetrance. Both alleles of c.855G>T were third codons of triplets coding for valine when the sequence was aligned with the published bovine *NUDT9* sequence, so only c.989G>A was a likely candidate for the cataract mutation. The *NUDT9* coding sequence of a single normal sheep was submitted to GenBank, HQ657210.

**Figure 4:**
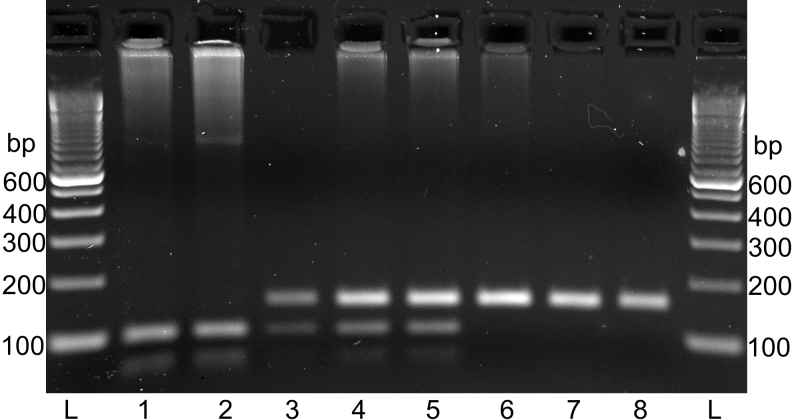
Restriction digest products. Animals with all three genotypes are represented in the samples. L indicates the 100 bp ladder. The other lanes by animal IDs, c.989G>A genotypes from sequencing, and cataract statuses are as follows: 1. 85/05, GG, normal; 2. 33/07, GG, normal; 3. 73/03, AG, affected; 4. 84/05, AG, affected; 5. 194/05, AG, affected; 6. 20/07, AA, affected; 7. 21/07, AA, affected; 8. 57/07, AA, affected.

Sequencing of both c.855G>T and c.989G>A in additional animals gave genotypes that were also correlated with cataract status, indicating that both polymorphisms were strongly linked to the cataract locus. The c.989G>A polymorphism was selected for genotyping by restriction digests in 141 additional animals because it was both associated with an amino acid change (arginine to histidine) and correlated with cataract status. These genotypes were consistent with those from direct sequencing (Figure 4).

The 238 c.989G>A genotypes showed a clear association with cataract status. A total of 163 out of 165 affected animals were A homozygotes or heterozygotes, whereas 44 out of 73 normal animals were G homozygotes. Two affected animals are G homozygotes, which is not consistent with c.989G>A being the cataract mutation, even allowing for incomplete penetrance.

The c.989G>A genotypes of the 81 lambs are shown in Table 5, along with their cataract statuses. Of the 81 predictions made, 84% were correct and 16% were incorrect. All of the errors were animals with the AG genotype that did not develop cataracts. Of the 62 animals that had either the AA or AG genotype, 79% (49) were affected.

**Table 5:** Cataract statuses and c.989>A genotypes.

**Cataract status**	**GG**	**AG**	**AA**	**Total**
Unaffected (expected)	19 (19)	13 (0)	0 (0)	32 (19)
Affected (expected)	0 (0)	32 (45)	17 (17)	49 (62)
Total	19	45	17	81

Results for 81 animals in the same birth cohort are shown.

## Discussion

Since OHC can be inherited from a single affected parent by both male and female animals, and there is no significant difference in cataract frequency between males and females, OHC is probably not X- or Y-linked and the most probable mode of inheritance is autosomal dominant. Therefore, the cataract locus is likely to be on one of the 26 sheep autosomes. A genome scan was performed using markers on 20 autosomes (equivalent to about 86% of the sheep genome), revealing significant linkage between the OHC locus and three markers on chromosome 6. It is possible that one or more loci on the remaining autosomes is linked to OHC, but as 80% of the heritability is due to the loci on chromosome 6, this would be difficult to detect with linkage mapping.

Assuming that OHC is an autosomal dominant trait and all affected parents were heterozygotes, the number of progeny in breeding trials is expected to have included about 65 animals homozygous for the cataract allele, but none have been identified. This may be due to each individual homozygote producing insufficient offspring to be identified, or incomplete penetrance which prevents the development of the cataract phenotype in some of the animals. Significantly fewer animals developed cataracts than would be expected from simple autosomal dominant inheritance, which also supports incomplete penetrance.

It is also possible that there is a lethal homozygote effect in OHC, which kills homozygous animals before they can be identified as affected, reducing the proportion of affected animals. In this study, the proportion of affected offspring from two affected parents is not significantly different from dominant inheritance with a lethal homozygote effect. With this mode of inheritance, offspring of two affected parents would either have smaller litter sizes or higher death rates before eye examination, compared to offspring of one affected and one normal parent.

Death rates are significantly higher in offspring of two affected parents versus one affected and one normal parent. However the excess death rate is too small to be evidence of a lethal homozygote effect, or to explain the deficiency in affected animals. A lethal homozygote effect also would not explain the deficit in affected animals with only one affected parent. Therefore it is unlikely that the inheritance of OHC involves a lethal homozygote effect, and the deficit in affected animals is probably due to incomplete penetrance.

**Figure 5:**
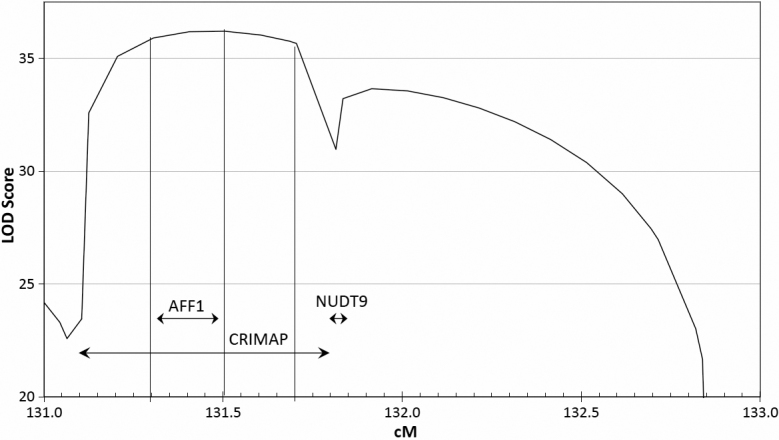
Close-up of Figure 3. The boundaries of the 1-LOD support interval and the point with the highest LOD score are shown by vertical lines as in Figure 3. CRIMAP shows most likely interval between two markers to contain the OHC locus, from the latest CRIMAP analysis using the same data as the multipoint analysis. *AFF1* and *NUDT9* show the boundaries of those genes, taken from the Oar1.0 physical map and interpolated to the linkage map.

Incomplete penetrance in OHC may indicate that other genes influence the cataract phenotype. Linkage analysis in this investigation was performed using a model that assumed that OHC had full penetrance, even though investigation of the inheritance of OHC strongly indicated incomplete penetrance. It has been shown [16] that using a model with full penetrance in this situation tends to reduce LOD scores and underestimate the size of linked regions. However false positives are not more likely, because that would require increasing LOD scores from non-significant to significant. Therefore significant linkage between markers and the trait locus is as reliable as with an accurate model. The effect is similar but much stronger with multipoint analysis [17], so the 1-LOD support intervals shown in Figure 3 and Figure 5 may not be reliable.

One of the markers used in the final genome scan was a SNP in the coding sequence of the gene *NUDT9*, c.989G>A. This polymorphism was originally a candidate for the cataract mutation since it correlated with cataract status. However, large scale genotyping found two animals with genotypes incompatible with this being the cataract mutation. No other SNPs were found in *NUDT9* coding sequence that changed the amino acid sequence and were correlated with cataract status, so it is unlikely that *NUDT9* is the gene responsible for OHC. However, it is possible that a mutation in the promoter or intron regions of *NUDT9* is the cataract mutation.

The SNP was close enough to the most likely position of the cataract locus and had enough variation to be used as an additional marker in the genome scan. Comparing c.989G>A genotypes to cataract phenotypes in 81 animals has shown that the SNP can also be used as a genetic test for the cataract with 84% accuracy. Allowing for a penetrance of 0.81, this is a reasonably accurate genetic test for OHC.

The cattle genome assembly was further advanced than the sheep equivalent at the time of this study. Therefore when a linked region was generated by the genome scan, the equivalent region of the cattle genome was searched for candidate genes for OHC. The similar synteny between the two genomes means that these genes would also be present in the linked region of the sheep genome. Genes were considered candidates if they had any association with cataract or any known role in the lens.

The most likely position for the OHC locus is between 131.1 and 131.8 cM from the centromere of sheep chromosome 6. The latest bovine genome sequence, Btau 4.0, contains seven genes in the equivalent region (Table 6). Of these genes, only *AFF1* has a known association with cataracts. *NUDT9* is known to be expressed in the human lens [8] and codes for an enzyme that hydrolyses ADP-ribose. Excessive amounts of ADP-ribose can directly cause protein damage that may lead to cataract [9], and may have the potential to disrupt calcium homeostasis [10], leading to activation of calcium-dependent proteases, but there is no evidence of cataracts being caused by these mechanisms in any species.

**Table 6:** Genes in the OHC-linked region.

**Gene**	**Megabases**
*PTPN13*	105.59–105.81
*SLC10A6*	105.82–105.85
*C4orf36*	105.88–105.89
*AFF1*	106.02–106.12
*KLHL8*	106.14–106.15
*HSD17B11*	106.30–106.35
*NUDT9*	106.39–106.43

Positions are given in megabases from the centromere on bovine chromosome 6.

*AFF1* codes for a transcription factor, the mouse homolog of which is associated with the robotic mouse phenotype [11]. Along with ataxia, this phenotype is characterized by cataracts in mice and is inherited in a dominant manner. OHC sheep do not exhibit ataxia, but since no other gene in this interval is associated with cataracts, *AFF1* is probably the best candidate for the OHC gene. The 1-LOD support interval from the multipoint analysis contains *AFF1* (Figure 5), also suggesting it as a candidate for the OHC gene. One of the affected sires is currently being sequenced. Any obvious loss of function mutations in this or other genes in the region will be followed up and reported in a later publication.
